# A Novel Video Tracking Method to Evaluate the Effect of Influenza Infection and Antiviral Treatment on Ferret Activity

**DOI:** 10.1371/journal.pone.0118780

**Published:** 2015-03-04

**Authors:** Ding Yuan Oh, Ian G. Barr, Aeron C. Hurt

**Affiliations:** 1 WHO Collaborating Centre for Reference and Research on Influenza, VIDRL, at the Peter Doherty Institute for Infection and Immunity, Melbourne, Victoria, 3000, Australia; 2 School of Applied and Biomedical Sciences, Federation University, Churchill, Victoria, 3842, Australia; 3 Melbourne School of Population and Global Health, University of Melbourne, Parkville, Victoria, 3010, Australia; University of Georgia, UNITED STATES

## Abstract

Ferrets are the preferred animal model to assess influenza virus infection, virulence and transmission as they display similar clinical symptoms and pathogenesis to those of humans. Measures of disease severity in the ferret include weight loss, temperature rise, sneezing, viral shedding and reduced activity. To date, the only available method for activity measurement has been the assignment of an arbitrary score by a ‘blind’ observer based on pre-defined responsiveness scale. This manual scoring method is subjective and can be prone to bias. In this study, we described a novel video-tracking methodology for determining activity changes in a ferret model of influenza infection. This method eliminates the various limitations of manual scoring, which include the need for a sole ‘blind’ observer and the requirement to recognise the ‘normal’ activity of ferrets in order to assign relative activity scores. In ferrets infected with an A(H1N1)pdm09 virus, video-tracking was more sensitive than manual scoring in detecting ferret activity changes. Using this video-tracking method, oseltamivir treatment was found to ameliorate the effect of influenza infection on activity in ferret. Oseltamivir treatment of animals was associated with an improvement in clinical symptoms, including reduced inflammatory responses in the upper respiratory tract, lower body weight loss and a smaller rise in body temperature, despite there being no significant reduction in viral shedding. In summary, this novel video-tracking is an easy-to-use, objective and sensitive methodology for measuring ferret activity.

## Introduction

Influenza is a highly contagious, respiratory disease that causes high morbidity and mortality worldwide. Typical symptoms of influenza infections range from fever, malaise, sore throat and muscular pain, to fatal pulmonary or cardiac complications [1]. The recent 2009 H1N1 pandemic caused a moderate death toll compared to previous pandemic events but the threat of an emerging pandemic virus remains high with human cases of highly pathogenic avian influenza (HPAI) H5N1 and H7N9 infections continuing to occur [2, 3]. In preparation for such a pandemic event, a better understanding of the disease mechanisms and effectiveness of current and novel interventions strategies is needed.

Central to these studies is the use of animal models to investigate viral pathogenesis [4], the effectiveness of novel vaccines [5] or antivirals agents [6], and other intervention strategies to reduce transmission [7, 8]. Ferrets are the preferred animal model to assess influenza virus infection, virulence and transmission as they display similar clinical symptoms, pathogenesis and antibody responses to those of humans [9], and can be readily infected with seasonal, pandemic or potentially pandemic influenza viruses such as H1N1pdm09, H5N1 and H7N9 [4, 10–13].

Assessment of influenza infection in ferrets to date has typically consisted of measurement of weight loss, temperature, clinical symptoms (such as lethargy and sneezing) and viral clearance from the nasal cavity [7, 14]. The measurement of ferret activity (lethargy) is normally assessed by a widely-used method first described by Reuman *et al* [15]. This system involves assigning ferrets an arbitrary score largely based on a pre-defined responsiveness scale for the animal (for example, defining score 0 as ferret with normal activity, 1 as reduced activity and 2 as inactive). Published ferret studies have used different scoring systems that have varied in either their scale or the criteria used [6, 7, 16, 17]. This type of activity scoring method is generally known as the ‘manual’ scoring method and has been more commonly used in ferret studies involving HPAI viruses such as H5N1 [8, 17–19], characterised by severe disease pathogenesis and extreme changes in ferret activity. However, there are many limitations to this methodology of manually scoring ferret activity including potential bias when comparing the effect of a treatment (if the scoring is not conducted in a ‘blinded’ manner), the need for experienced personnel to recognise and assign activity scores relative to the ‘normal’ activity of a ferret, difficulty assessing activity within a small cage, and the broad range of scoring levels which may fail to capture subtle changes in activity. Many of these deficiencies can be overcome by the use of EthoVision XT video-tracking software which has been widely-used in studies of neurological and behaviour patterns in experimental animals such as rodents [20] and zebrafish [21]. Tracking relies on the analysis of the movement of the animal within the area of interest to measure parameters such as activity, distance travelled and velocity. In ferrets, video-tracking has been reported for investigating behaviour post-anaesthesia [22] but, to the best of our knowledge, video-tracking has never been used to examine the activity of ferrets in a model of infectious disease. In this study, we assessed the use of video-tracking analysis as an alternative method to manual scoring of ferret activity following influenza infection. In addition we used this novel analysis tool to investigate whether treatment with the antiviral drug, oseltamivir of influenza infected ferrets significantly altered their activity levels.

## Materials and Methods

### Ethics statement

Experiments using ferrets was conducted with approval from the CSL Limited/Pfizer Animal Ethics Committee (project license number 868) in strict accordance with the Australian Government, National Health and Medical Research Council Australian code of practice for the care and use of animals for scientific purposes (8^th^ edition). Animal studies were conducted at CSL Limited using services provided under a Support Service Agreement between CSL Limited and WHO Collaborating Centre for Reference and Research on Influenza.

### Ferrets

Adult male and female ferrets weighing 700–1300 g were used. Serum samples from ferrets were tested by hemagglutination inhibition (HI) assay against the reference strains A/California/7/2009 A(H1N1)pdm09, A/Victoria/361/2011 (H3N2), B/Wisconsin/1/2010 (B/Yamagata-lineage) and B/Brisbane/60/2008 (B/Victoria-lineage) to ensure seronegativity against currently circulating influenza subtypes and lineages.

### Virus infection and oseltamivir treatment of ferrets

Ferrets were anaesthetized [50:50 mix of Ketamine (100 mg/mL): Ilium Xylazil (Xyalazine; 20 mg/mL)] and infected artificially by intranasal inoculation with either 1×10^3^ or 1×10^5^ TCID_50_ (median tissue culture infectious dose) of MDCK (Madin-Darby canine kidney (MDCK; ATCC CCL-34)-propagated A/Perth/265/2009 A(H1N1)pdm09 (2.0 × 10^6^ TCID_50_/mL). A 5 mg/kg dose of oseltamivir phosphate in ferrets has been considered to be equivalent (based on weight) to the standard human adult dose of 75 mg, which is normally delivered twice daily for treatment [23]. Oseltamivir phosphate (kindly provided by Hoffmann-La Roche Ltd., Basel, Switzerland) was prepared at a concentration of 10 mg/mL in a sterile 0.5% (v/v) sugar/phosphate-buffered solution (PBS) solution and 5 mg/kg was delivered orally to conscious ferrets twice daily with the volume adjusted based on weight (e.g. 1200 g ferret received 600 μL). Ferrets were administered oseltamivir twice daily starting with the first dose 2 hours prior to infection. All oseltamivir treatment was carried out for a total of 5 days. Ferrets were housed individually in high efficiency particulate air filtered cages with free access to food, water and enrichment equipment such as toys throughout the experimental period. A single room was used exclusively to house the ferret cages (n = 12) for this study to maintain minimal disturbance from the main animal holding facility. Infected ferrets were observed for a 10-day period with the infection day of all experiments occurring on a Monday (day 0 post-infection) and the last day of the experiment on a Friday (day 11 post-infection).

### Ferret monitoring, measurement and sample collection

Body temperature, weight and nasal washes of all ferrets were collected daily post-infection. Temperatures were measured daily using implanted temperature transponders fitted to identification chips (LifeChip Bio-Thermo, Digivet, Australia). Nasal washes were collected daily from sedated ferrets (intramuscular injection of Xylazine at 5 mg/kg) by instilling 1 mL of sterile PBS into one nostril and allowing the liquid to flow out of the other nostril into a collection tube. The number of cells in the nasal washes was counted immediately after collection. Aliquots of nasal washes were stored at -80°C prior to determining the virus titres and protein concentrations. Blood samples were collected 10 days post-infection and sera stored at -80°C prior to testing for influenza-specific antibodies. Ferrets were sacrificed 10 days post-infection by intramuscular injection of anaesthesia [50:50 mix of Ketamine (100 mg/mL): Ilium Xylazil (Xyalazine; 20 mg/mL)] followed by an overdose of pentobarbitone sodium (Lethabarb; 0.5 mL/kg).

### Inflammatory, virological and serological analysis

Inflammatory cell count in the nasal wash was determined by Trypan blue exclusion using a Countess automated cell counter (Life technologies, Australia). Protein concentration in the nasal wash was determined using Coomassie Plus (Bradford) assay as according to the manufacturer’s instructions (Thermoscientific, Australia). Titres of infectious virus in the nasal washes were quantified by viral infectivity assay and a TCID_50_ determined [24]. Briefly, samples were serially diluted 10-fold in PBS and each dilution added in triplicate to flat-bottom 96-well plates containing a confluent monolayer of MDCK cells. Infected cells were incubated for 4 days at 35°C, 5% CO_2_ followed by detection of virus by addition of 25 μL of 1% Turkey red blood cells (RBCs) to 25 μL of infected cell supernatant (wells containing fully hemagglutinated RBCs were scored as positive for influenza virus). Virus titres were calculated as described by Reed and Muench [25]. The influenza-specific antibodies in sera were assessed by HI assay [26]. Briefly, receptor destroying enzyme (RDE; Denka Seiken, Japan)-treated sera were serially 2-fold diluted from a starting dilution of 1:20 in V-bottom 96-well plates. Virus (4 hemagglutination units) was added to all wells and 1% Turkey red-blood cells were added after one hour incubation at room temperature. Positive wells were defined as those where there was complete inhibition of hemagglutination.

### Activity measurement: manual scoring

Manual activity scoring of individually housed ferrets was performed in a ‘blinded’ manner at the same time each day by an experienced animal technician. A seven-level arbitrary activity scale was used according to a previously published study of A(H1N1)pdm09 virus [7]: 0: alert and fully playful; 0.5: alert but slightly less playful than usual; 1: alert and playful when encouraged to play; 1.5: alert and slightly playful when encouraged to play; 2: alert and slightly playful with strong encouragement; 2.5: alert but not playful, and 3: neither alert nor playful (required to be euthanized). Ferrets were ‘encouraged’ to play by firstly tapping the cage window gently to gain their attention, followed by wiggling the technician’s fingers as an encouragement to play. All attempts of encouragement were carried out while the ferrets were in the cages. Manual activity scoring of the ferrets was carried out at least 3 hours after filming to minimise unnecessary disturbance to the ferrets which may affect either activity scores. Mean activity scores for ferrets in each experimental group were calculated as described previously [16].

### Activity measurement: video filming and software analysis

Video analysis of ferret activity was assessed daily at the same time by placing a single ferret into a 400 litre plastic box (length 97.5cm × width 75.5cm × height 67.6cm; Theplasticman, Australia; referred to as the ‘activity box’) and recording movement for 1 to 5 minutes after 7 seconds of acclimatisation on a video camera (MAGINON digital full HD camcorder; Supra, Germany) positioned above the box. Longer acclimatisation periods (1, 2, 3 and 4 minutes), prior to commencing filming decreased the overall activity rate with each additional minute of acclimatisation and thus 7 seconds was chosen as the optimal acclimatisation time (S1 Fig.). Relative to the size of a typical ferret, the 400 litre box was considered a better option than a 100 litre box as there was a larger area for movement and therefore we would expect a more accurate activity measurement (S2 Fig.). The interior of the 400 litre box was painted grey matt (colour code 3102; PlastiKote, USA) to enable a good contrast between both the white and dark-coloured ferrets used in the experiments, and the background colour. Prior to painting, the box was gloss white in colour which was highly reflective and resulted in reflections of the animal on the side wall of the box which misled the video analysis software and led to inaccurate activity measurements. Between the filming of each ferret, the box was decontaminated with 80% (v/v) ethanol to remove residual smell and/or viral contamination from the previous ferret. This cleaning protocol was effective in preventing viral transmission from one ferret to another [non-infected ferrets remained infection-free (absence of virus shedding and influenza-specific antibodies) even when they were filmed directly after an infected ferret for 10 consecutive days]. The filming sequence of individual ferrets in all groups was randomly assigned and filming was carried out once in the morning prior to other procedures such as oseltamivir dosing or nasal washing.

### EthoVision settings for activity, distance and velocity analyses

Activity (pixel change), distance moved (m) and velocity (m/s) were measured using an automated video-tracking and motion analysis program, EthoVision XT 10.0 (Noldus IT, Netherlands). Video files (AVI. format, filmed at 30 frames/second, 640 × 480 resolution) were uploaded into the program and analysed for activity, distance and velocity using program settings as outlined in Table 1. Program settings for activity measurement included ‘gray scaling’ as the detection method, and activity threshold and noise filter adjusted to 6 and 2 respectively to achieve minimal background noise and zero activity detection when the animal was stationary (Table 1; S1 Video). Activity level of ferrets was determined by the software as a function of pixel changes, where ferret movement results in pixel changes as represented by the purple colour pixels in S1 Video. Specific measurement of pixel changes involved analysing video films at a sampling rate of 30 samples/second (as the maximum rate for video filmed at 30 frames/second). The gray scale of each pixel of the film image was compared from one sample to the next, and the proportion of pixel changes was calculated (the number of pixels where the gray scale has changed from one sample to the next divided by the overall number of pixels in the image). An overall mean pixel change was finally determined by taking an average of pixel changes in the total samples within the film period (e.g. 1800 samples per minute of film). To investigate how the sampling rate may affect the mean activity result, we compared the maximum rate (30 samples/second) with two slower rates (15 samples/second and 1 sample/second) in the same set of ferret films. The maximum sampling rate was found to be the most sensitive, resulting in the greatest number of days where ferret activity was determined to be significantly different from baseline levels following influenza infection (S3 Fig.). To accurately locate both white and dark-coloured ferrets for distance and velocity analyses (the program automatically locates the centre point of the animal as represented by the red dot in S2 Video and tracks the distance and velocity), ‘differencing’ was used as the other detection methods (static subtraction, dynamic subtraction and gray scaling) were not able to detect the ferret and distinguish ferrets with different coloured coats. Sensitivity settings for ‘differencing’ were adjusted to 17 with subject size between 159 and 14436 for optimal animal detection (Table 1; S2 Video).

**Table 1:** Program settings for measuring activity, distance and velocity in EthoVision software.

Settings		Measurements		
		Activity	Distance	Velocity
Method		Gray scaling	Differencing	Differencing
Detection sensitivity		255	17	17
Subject size	Min	NA	159	159
	Max	NA	14436	14436
Video sample rate		30.1159/sec	30.0987/sec	30.0987/sec
Smoothing set-up		None	LOW	LOW
Track noise reduction		ON	ON	ON
Activity threshold		6	NA	NA
Background noise filter		2	NA	NA
Compression artefacts filter		OFF	NA	NA
Subject contour	Contour erosion	1 pixel	1 pixel	1 pixel
	Contour dilation	1 pixel	1 pixel	1 pixel

NA: Not applicable

### Statistical analyses

Mann-Whitney U non-parametric test was used to compare day-to-day differences in mean activity, distance, velocity and body temperature to mean baseline value. Kruskal-Wallis non-parametric test was used to compare nasal wash cell concentration, protein concentration, percentage weight loss and virus titre among the different groups. A P-value *of* <0.05 was considered statistically significant.

## Results

### Optimising activity measurement: Filming duration

**Fig 1:**
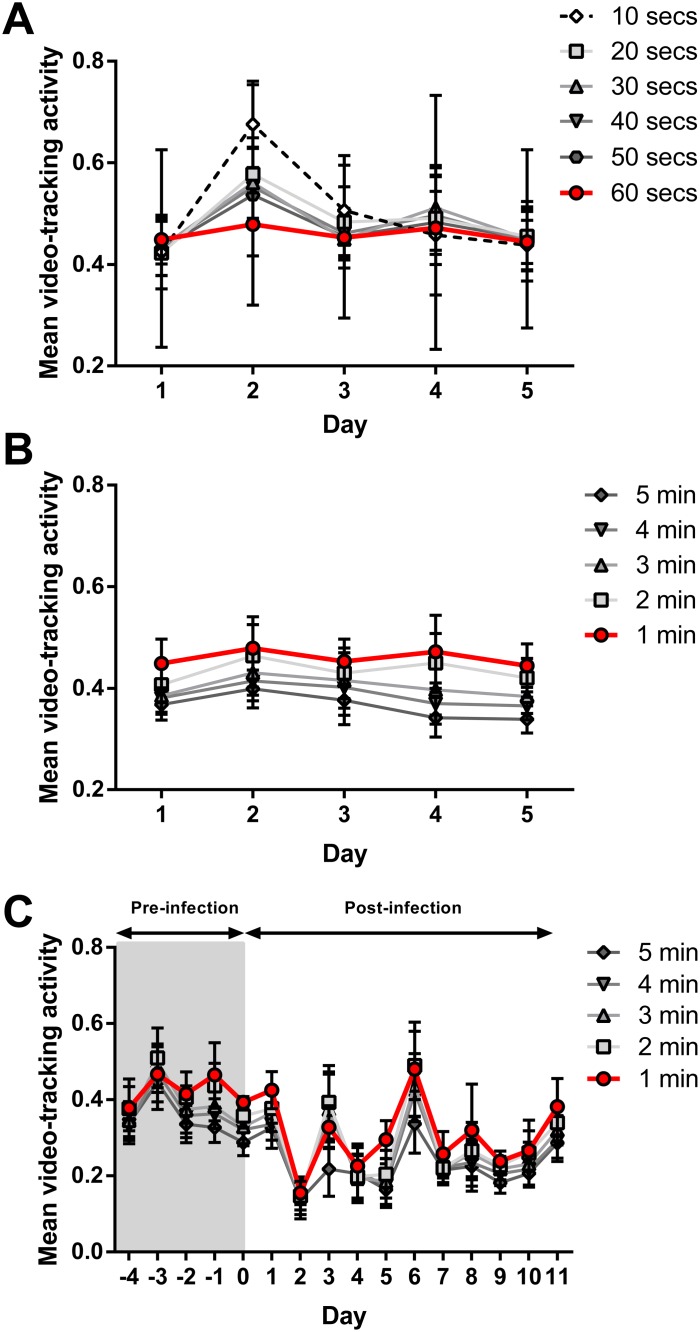
Optimal filming duration for ferret activity analysis. Ferrets were filmed daily for a total duration of 5 minutes on 5 consecutive days and mean activity analysed using EthoVision XT. Mean activity analysis of filming for periods of (A) 10 to 60 seconds and (B) 1 to 5 minutes of non-infected ferrets (n = 12). (C) Mean activity analysis of filming for periods of 1 to 5 minutes of ferrets intranasally-infected with 10^3^ TCID_50_ (500 μL; 250 μL per nostril) A(H1N1)pdm09 (n = 4). Data shown are mean±SEM.

Prior to measuring the effect of influenza infection on ferret activity, it was important to understand how activity changes over time in non-infected ferrets and to determine the video filming duration that resulted in the highest activity readings with the lowest day-to-day variability. Activity level of the ferrets was assessed as a function of pixel changes over time by the analytical software as described in the materials and methods. Non-infected ferrets were filmed in the activity box for 5 minutes each day for 5 days and the activity was calculated following analysis of different durations of film starting from time zero (S1 Video). Analysis of 60 seconds of film resulted in data that was less variable over the 5 day period compared to shorter filming durations (10, 20, 30, 40 and 50 seconds) (Fig. 1A). Analysis of film durations of more than 1 minute showed that activity, following analysis of 1, 2, 3, 4 or 5 minutes film, was equally stable across the 5 day period, but the overall activity rate decreased with each additional minute of film analysis (analysis of 1 minute of film resulted in the highest mean activity level whereas analysis of 5 minutes of film gave the lowest activity level) (Fig. 1B). Similarly, 1 minute filming was also shown to be the optimal duration when measuring the activity of ferrets post-infection with influenza (Fig. 1C). Therefore, 60 seconds was selected as the optimal duration of film analysis for all subsequent experiments.

### Effect of oseltamivir treatment on activity of influenza infected ferrets

**Fig 2:**
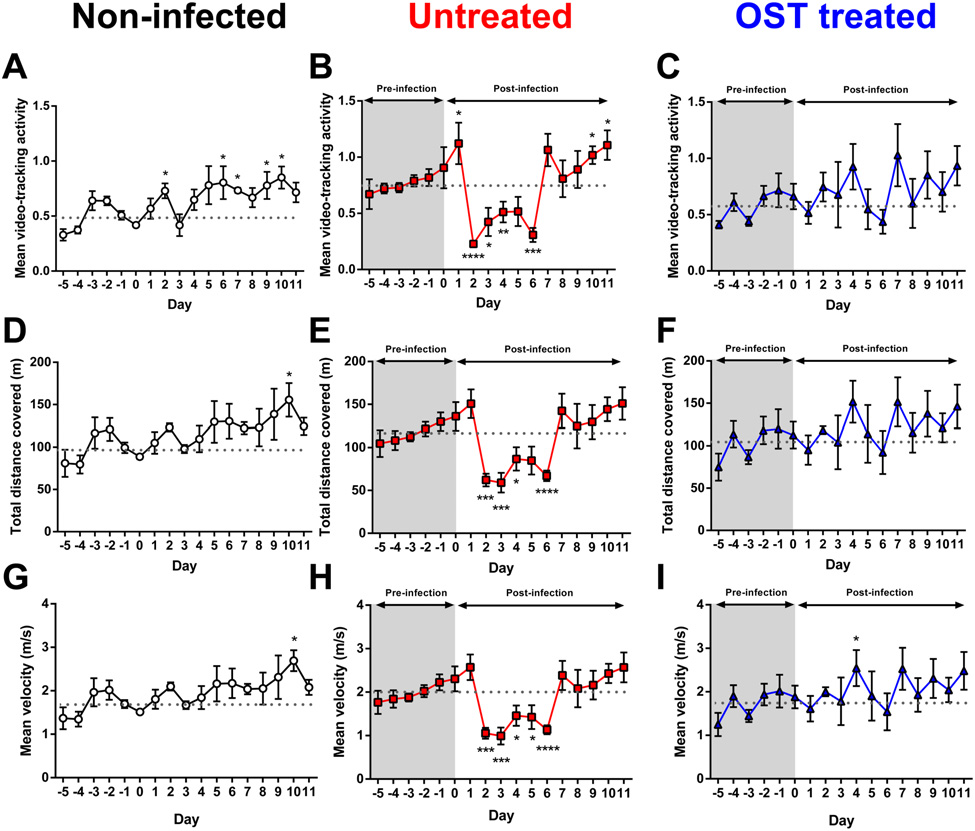
Oseltamivir treatment ameliorates the effect of influenza infection on ferret activity. (A—C) Mean activity, (D—F) total distance covered and (G—I) mean velocity of non-infected ferrets (n = 3), untreated ferrets (n = 4) and oseltamivir (OST)-treated ferrets (n = 4). Untreated and OST-treated ferrets were both intranasally-infected with 10^3^ TCID_50_ (500 μL; 250 μL per nostril) A(H1N1)pdm09. Only OST-treated ferrets were given sugar solution. Data shown are mean±SEM. Dotted line is the baseline average of the daily activity level prior to infection on day 0. Mean versus baseline average: *P<0.05, **P<0.01, ***P<0.001, ****P<0.0001, Mann-Whitney U test.

To determine the effect of oseltamivir treatment on ferret activity, animals were given a prophylactic dose of 5 mg/kg oseltamivir two hours prior to infection and thereafter twice daily for five days. Video-tracking analysis was used to assess ferret activity as well as distance travelled and velocity (S1 and S2 Videos). Influenza-infected ferrets with no intervention and non-infected ferrets were included as positive and negative controls respectively. In non-infected ferrets, the activity, distance and velocity of the animals from days 1 to 11 was equivalent to, or on some days higher than, the baseline average from days-5 to day 0 (mean activity: 0.49±0.03; total distance covered: 98.7±5.7; mean velocity: 1.66±0.09) (Fig. 2A, D and G). In infected ferrets that were not treated with oseltamivir, the activity level was significantly reduced (0.22±0.02; P<0.0001) on day 2 post-infection (pi) and remained significantly lower than the baseline (0.77±0.04) for four further days, before returning to baseline level or higher on day 7 (Fig. 2B). Both distance covered and velocity were also significantly lower than baseline in the untreated ferrets from day 2 to day 6, before returning to a level similar to that seen prior to infection (Fig. 2E and H).

In comparison, the administration of oseltamivir 2 hours prior to infection and twice daily for 5 days, ameliorated the impact of influenza infection on activity level (Fig. 2C, F and I). Similar to non-infected ferrets, the activity, distance moved and velocity of the oseltamivir-treated ferrets remained relatively similar to the pre-infection baseline average (mean activity: 0.58±0.04; total distance covered: 105.5±6.7m; mean velocity: 1.75±0.10m/s) (Fig. 2C, F and I). To check these findings, we repeated the experiment using a higher dose of virus inoculum [10^5^ TCID_50_ influenza A(H1N1)pdm09 virus] and obtained similar results, with untreated ferrets experiencing 4 to 6 days of significantly lower activity, distance covered or velocity following infection, whereas oseltamivir-treated ferrets displayed no significant change in any of these parameters (S4 Fig.).

### Comparison of video activity with manual scoring for assessing ferret activity

**Fig 3:**
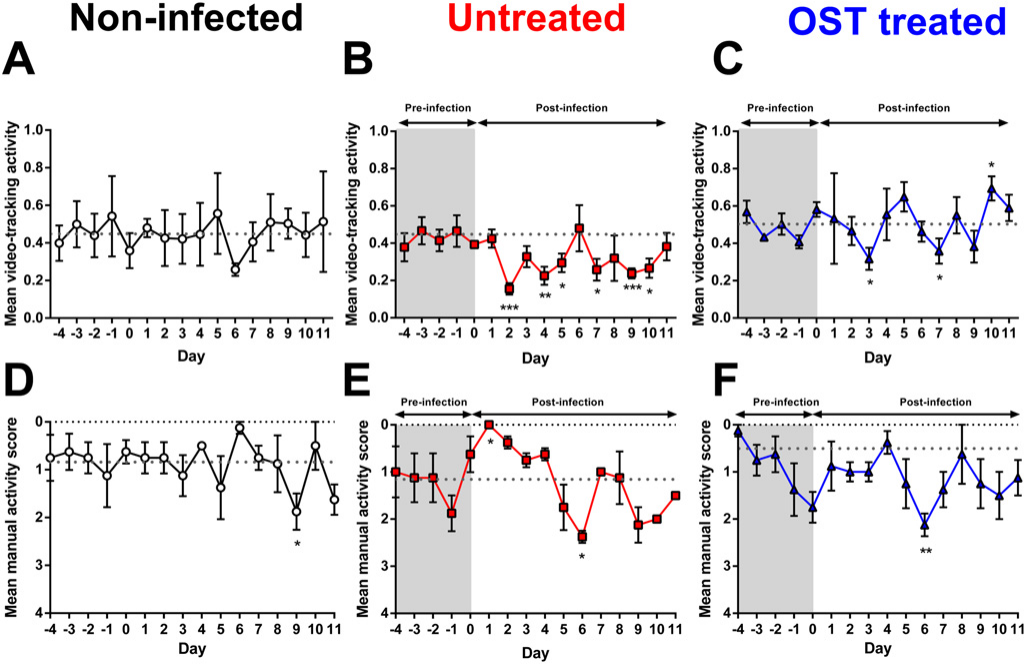
Comparison of video-tracking with manual scoring for assessing ferret activity. (A—C) Video-tracking analysis and (D—F) manual scoring of the mean activity of non-infected ferrets (n = 4), untreated ferrets (n = 4) and oseltamivir (OST)-treated ferret (n = 4). Untreated and OST-treated ferrets were both intranasally-infected with 10^3^ TCID_50_ (500 μL; 250 μL per nostril) A(H1N1)pdm09. Untreated ferrets were given sugar solution as a treatment control. Data shown are mean±SEM of an experiment independent to Fig. 2. Dotted line is the baseline average of the daily activity level prior to infection on day 0. Mean versus baseline average: *P<0.05, **P<0.01, ***P<0.001, Mann-Whitney U test.

To compare video-tracking with manual scoring, we repeated the infection experiment as outlined above and assessed the activity of ferrets using both methods. Activity of the ferrets over the 5-day pre-infection period was less variable using video-tracking analysis than using manual scoring method (Fig. 3). In the video-tracking analysis, the activity of non-infected ferrets from days 1 to 11 was equivalent to the baseline average from days-5 to day 0 (Fig. 3A). Similar to Fig. 2B, ferrets that were not treated with oseltamivir exhibited a significant drop in activity following viral infection (compared to pre-infection baseline data) on days 2, 4, 5, 7, 9 and 10 (Fig. 3B). As for oseltamivir-treated ferrets, the activity level remained relatively similar to the pre-infection baseline average except on day 3, 7 and 10 (Fig. 3C).

Using manual scoring carried out on the same groups of animals, the activity of non-infected ferrets were equivalent to the baseline average on most days, except on day 9 (Fig. 3D). For ferrets that were not treated with oseltamivir, we saw a significant increase in ferret activity on day 1 and a significant decrease in activity on day 6 (compared to the baseline data), with activity on all other days being similar to the pre-infection baseline (Fig. 3E). Oseltamivir-treated ferrets showed a similar levels of activity to baseline average for most days except on day 6 where there was a significant decrease in activity compared to baseline average (Fig. 3F). Importantly, the mean activity score of the post-infection period (a widely-used method for manual activity scores) of oseltamivir-treated ferrets was not significantly different to untreated ferrets (untreated: 1.19±0.14 vs. oseltamivir-treated: 1.19±0.24; P = 0.828) (Table 2). Therefore, following the comparison of the two methods, video-tracking methodology appeared to be more sensitive in detecting changes in ferret activity than the manual scoring method.

**Table 2:** Mean manual activity score of non-infected ferrets and infected ferrets with or without oseltamivir treatment from day 0 to day 11 post-infection.

Group	Non-infected (Control)	Untreated	OST treated
Mean manual activity score	0.91 ± 0.24	1.19 ± 0.14	1.19 ± 0.24

OST: Oseltamivir

### Effect of oseltamivir on the inflammatory and virological responses in influenza infected ferrets

**Fig 4:**
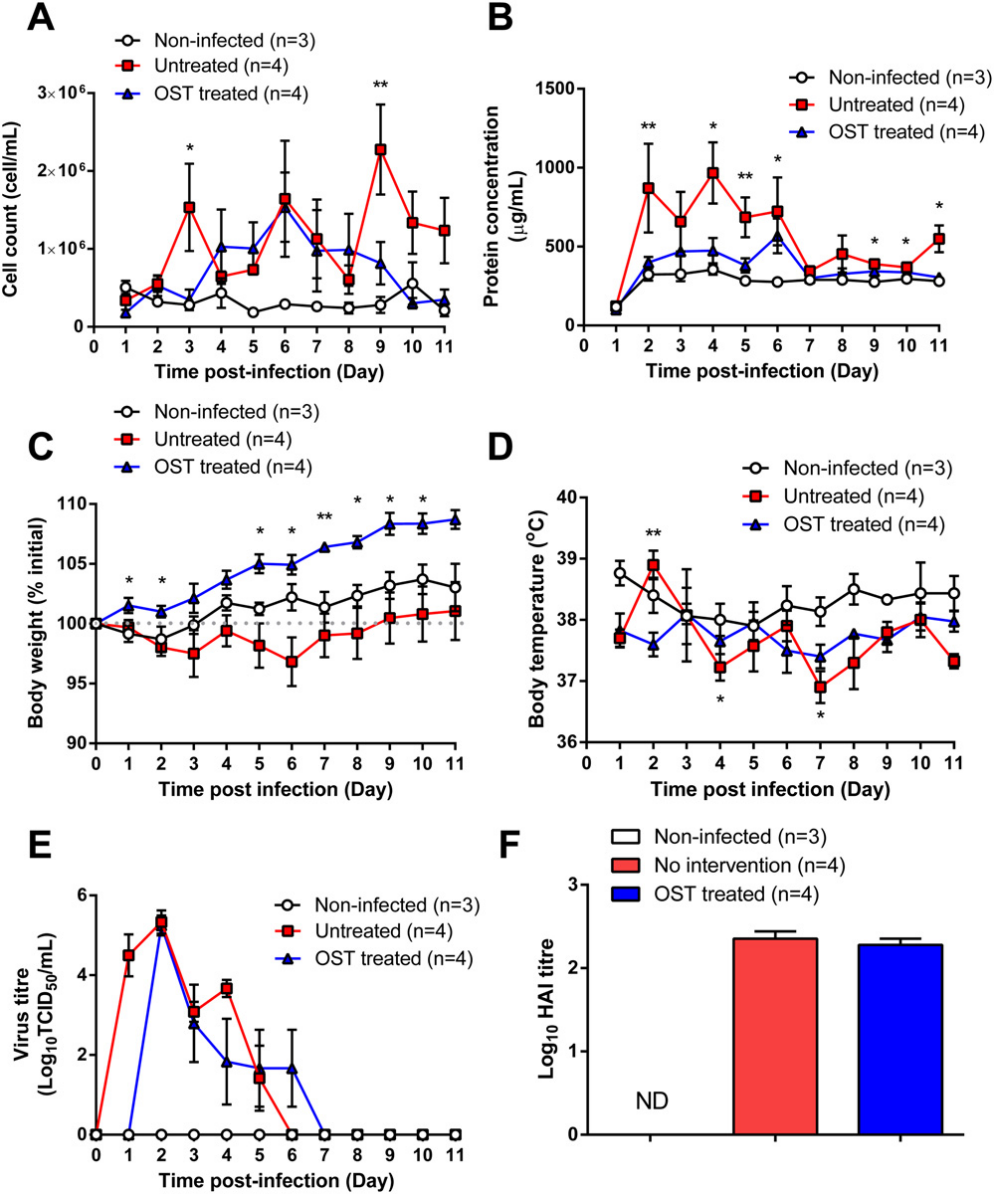
Oseltamivir treatment improves clinical symptoms of infected ferrets despite no reduction in viral load. (A) Cell number and (B) protein concentration in nasal washes. (C) Body weight. (D) Body temperature; mean temperature versus day 0 temperature: *P<0.05, **P<0.01, Mann-Whitney U test. (E) Virus titre in nasal wash. (F) Serum antibody titre. ND: Non-detected. OST: Oseltamivir. Data shown are mean±SEM from the same experiment in Fig. 2. Data comparison in (A—C): *P<0.05, **P<0.01, Kruskal-Wallis test.

Given the significant change in activity following oseltamivir treatment, it was important to investigate the inflammatory and virological changes in the ferrets. In untreated ferrets, the total cell concentration in nasal washes was significantly higher at day 3 and day 9 pi than in oseltamivir-treated or non-infected ferrets (Fig. 4A). The nasal wash of untreated ferrets also contained a significantly higher concentration of protein at day 2, 4, 5, 6, 9, 10 and 11 pi compared to oseltamivir-treated and non-infected groups, which remained relatively similar over the post-infection period (Fig. 4B). Untreated ferrets lost weight from day 1 pi before starting to regain weight after day 6 pi (Fig. 4C). In contrast, oseltamivir-treated ferrets had a steady increase in body weight from day 1 pi and were significantly higher compared to untreated and non-infected ferrets for most of the experimental period (Fig. 4C). Untreated ferrets showed significant increases in body temperature from baseline temperature at day 2 pi (day 0: 37.7±0.15°C; day 2 pi: 38.9±0.23°C; P = 0.007), whereas no significant changes in body temperature were observed for oseltamivir-treated and non-infected ferrets (Fig. 4D). However despite the significant improvement in morbidity as a result of oseltamivir treatment, there was no clear reduction in either the peak viral load (log_10_TCID_50_/mL: untreated: 5.33±0.29; oseltamivir-treated: 5.25±0.25; P>0.99) or virus shedding duration (untreated: 5 days; oseltamivir-treated: 5 days) (Fig. 4E). There was a one day delay in viral shedding in oseltamivir-treated ferrets compared to untreated ferrets. No significant difference in HI antibody titre was observed between the untreated and oseltamivir-treated groups when examined at day 10 pi (Fig. 4F).

## Discussion

In this study we have shown that video-tracking analysis was sufficiently sensitive and reproducible to detect changes in ferret activity following A(H1N1)pdm09 infection and to show positive effects of oseltamivir on maintaining the activity of influenza-infected animals. Oseltamivir treatment was also associated with improved clinical symptoms including reductions in inflammatory responses in the upper respiratory tract, lower body weight loss and a smaller body temperature rises following influenza infections, despite there being no reduction in viral shedding in the ferret nasal wash samples.

To date, the only methodology used to measure ferret activity has been the manual assignment of an arbitrary score based on a pre-defined scale of responsiveness [6–8, 15–17]. Based on our experience, one of the difficulties of using this method is the requirement for the observer to recognise the ‘normal/baseline’ activity level of individual ferrets (considering the inherent large ferret-to-ferret variation in activity) which becomes challenging when large numbers of ferrets are used in a typical experiment. The majority of existing manual scoring methods encompass a scale of responsiveness that only measures loss of activity (compared to ‘normal’), whereas the video-tracking method measures both increases and decreases of activity compared to a pre-infection baseline [6, 7, 16, 17]. In addition, manual scoring provides only a relative activity comparison, as it relies on defining the pre-infection behaviour of each ferret as ‘normal’ (score 0), regardless of the inherent difference in activity between different ferrets. This means that two ferrets with the same manual score (e.g. 1) may have considerably different absolute activities. In comparison, the output from the video-tracking method yields a value which allows both relative comparisons and absolute activity comparisons between different ferrets which should make activity measurements more accurate in each experiment.

Manual scoring is also subjective as it depends on the discretion and experience of the observer to assign a score that best describes the ferret activity and therefore, restricts the scoring process to a single observer to allow accurate comparisons of activity within and across experiments. Additionally, the requirement to score the ferret while it is still in the cage may bias the results, for example, healthy ferrets may be scored as less active due to being housed in single isolation cage compared to situations when there are other ferrets to play with. In contrast, the advantage of video-tracking methodology is that it removes the need for a ‘blind’ observer which means that the whole process, from filming to software analysis, can be carried out by a single person. Additionally, there is no need to have knowledge of the individual ferrets ‘normal’ activity (as needed in manual scoring for assigning a relative score), allowing anyone to conduct the filming. Finally, the ability to measure multiple parameters such as activity, distance moved and velocity in video-tracking analysis will provide a better assessment of the activity state of the infected ferrets compared to the single parameter manual scoring method. A potential limitation of this study is that ferrets are filmed or viewed only once per day, therefore providing only a snap-shot of the ferret activity. If ferret activity fluctuated significantly throughout the day it is possible that incorporating more filming/viewing time-points may be required.

In our previous study, we have shown that manual scoring can detect a difference in ferret activity following influenza A(H1N1)pdm09 virus infection [7]. However, other studies reported only minimal changes post-infection with influenza viruses such as A(H1N1)pdm09 (A/California/04/2009) and A(H3N2) (A/Brisbane/10/2007) when using manual ferret activity scoring [6, 16, 17]. In this study, manual scoring appeared to be less sensitive than video-tracking analysis in detecting changes in activity. Collectively, these findings suggest that video-tracking analysis may be better suited than the manual scoring method for detecting activity changes associated with seasonal virus infection, which generally induces only a mild disease.

Previously, studies which used the manual scoring method have reported different findings on the effect of oseltamivir on ferret activity scores [6, 16]. Govorkova *et al* [6] reported that following infection with influenza A/California/4/2009, oseltamivir-treated ferrets showed improved activity (mean score of ~0) compared to untreated ferrets (mean score of 1). Using the same virus, Marriott *et al* [16] reported significant improvement in activity of ferrets given oseltamivir treatment compared to no improvement when given as prophylaxis. However, none of these studies showed the activity changes throughout the infection period. Consistent with the findings of Marriott *et al* [16], we observed a one day delay in detectable virus shedding in the oseltamivir treated group with no difference in either viral shedding duration or peak viral load compared to ferrets with no intervention. By video-tracking, we confirmed that ferrets treated with oseltamivir still shed considerable amounts of virus at the same time as showing an improvement in activity level. As highlighted in our previous publication [7], this finding is of significant public health concern with implications for the management of viral transmission by oseltamivir-treated patients who feel ‘well’ but are still shedding virus.

The use of ferrets as an *in vivo* model of infection is not limited to influenza virus alone and includes other viruses of public health concern such as Hendra virus [27], Nipah virus [28] and severe acute respiratory syndrome-coronavirus (SARS-CoV) [29]. With additional testing to validate its applicability to infections with other viruses, this improved method for monitoring ferret activity has the potential to assist in measuring both the pathogenesis and the impact of therapeutic interventions such as vaccines and antiviral drugs using the ferret model of infection.

## Supporting Information

S1 Fig Optimal acclimatisation time prior to ferret activity analysis. Video-tracking analysis of the mean activity of non-infected ferret (n = 4) for a total duration of 5 minutes on 5 consecutive days using EthoVision. Ferrets were left in the box for different time period (7 seconds, 1, 2, 3 and 4 minutes) prior to 1 minute of filming. Data shown are mean±SEM.
(EPS)

S2 Fig Comparison of the different sizes of the activity box used for filming activity measurements. (EPS)

S3 Fig Optimal frame rate for video-tracking analysis in EthoVision XT. Video-tracking analysis of the mean activity of non-infected ferret (n = 4), untreated ferrets (n = 4) and oseltamivir (OST)-treated ferret (n = 4) for 1 minute at different frame rate of 1, 15 and 30/seconds. Untreated and OST-treated ferrets were both intranasally-infected with 10^3^ TCID_50_ (500 μL; 250 μL per nostril) A(H1N1)pdm09. Only OST-treated ferrets were given sugar solution. Data shown are mean±SEM of the same experiment in Fig. 2. Dotted line is the baseline average of the daily activity level prior to infection on day 0. Mean versus baseline average: *P<0.05, **P<0.01, ***P<0.001, ****P<0.0001, Mann-Whitney U test.
(EPS)

S4 Fig Oseltamivir treatment ameliorates the effect of influenza infection on ferret activity with a higher virus inoculum of 10^5^ TCID_50_. (A—C) Mean activity, (D—F) total distance covered and (G—I) mean velocity of non-infected ferrets (n = 3), untreated ferrets (n = 4) and oseltamivir (OST)-treated ferrets (n = 4). Untreated and OST-treated ferrets were both intranasally-infected with 10^5^ TCID_50_ (500 μL; 250 μL per nostril) A(H1N1)pdm09. Only OST-treated ferrets were given sugar solution. Data shown are mean±SEM. Dotted line is the baseline average of the daily activity level prior to infection on day 0. Mean activity versus baseline average: *P<0.05, **P<0.01, ***P<0.001, Mann-Whitney U test.
(EPS)

S1 Video Representative video-tracking analysis of the activity of a non-infected ferret. Purple pixels represent movement of ferret in the activity box.
(MP4)

S2 Video Representative video-tracking analysis of the distance and velocity of a non-infected ferret. Red dot represent the centre point of the animal located automatically by the program and the red line represent the moving tracks of the ferret.
(MP4)
